# White matter anisotropy and response to cognitive behavior therapy for posttraumatic stress disorder

**DOI:** 10.1038/s41398-020-01143-3

**Published:** 2021-01-05

**Authors:** Mayuresh S. Korgaonkar, Kim L. Felmingham, Aleksandra Klimova, May Erlinger, Leanne M. Williams, Richard A. Bryant

**Affiliations:** 1 Brain Dynamics Centre, Westmead Institute for Medical Research, The University of Sydney, Sydney, NSW Australia; 2 School of Health Sciences, Faculty of Medicine and Health, The University of Sydney, Sydney, NSW Australia; 3 School of Psychological Sciences, University of Melbourne, Melbourne, VIC Australia; 4 Department of Psychiatry and Behavioral Sciences, Stanford University, Stanford, CA USA; 5 Sierra-Pacific Mental Illness Research, Education and Clinical Center (MIRECC) VA Palo Alto Health Care System, Palo Alto, CA USA; 6 School of Psychology, University of New South Wales, Sydney, NSW Australia

**Keywords:** Predictive markers, Human behaviour

## Abstract

Trauma-focused cognitive behavior therapy (TF-CBT) is the gold standard treatment for posttraumatic stress disorder (PTSD), up to one-half of PTSD patients remain treatment non-responders. Although studies have used functional MRI to understand the neurobiology of treatment response, there is less understanding of the role of white matter brain structures in response to TF-CBT. Thirty-six treatment-seeking PTSD patients and 33 age-gender matched healthy controls completed diffusion-weighted imaging scans at baseline. Patients underwent nine sessions of TF-CBT treatment and PTSD symptom severity was assessed with the Clinician-Administered PTSD Scale before and after completing treatment. Patients were assessed to estimate the reduction in overall symptoms and also specifically fear and dysphoric symptoms of PTSD. Tract-based spatial statistical analyses were performed for the PTSD group to evaluate whole-brain correlations of fractional anisotropy (FA) with improvement in overall, fear, and dysphoric symptoms using non-parametric permutation inference testing (*p*_FWE_ < 0.05). Next, we evaluated if these significant measures also characterized PTSD from controls. Greater improvement in dysphoric symptoms was found correlated with lower FA in white matter regions associated with the limbic system, frontal cortex, thalamic association and projection fibers, corpus callosum, and tracts related to the brainstem. White matter anisotropy was not found associated with either overall or fear symptoms. FA in the significant clusters was similar between PTSD and controls. White-matter related to key functional regions may also play an important role in response to TF-CBT. Our results underscore the heterogeneity of PTSD and the need to evaluate distinct symptom phenotypes in treatment studies.

## Introduction

International treatment guidelines recommend trauma-focused cognitive behavior therapy (TF-CBT) as the recommended treatment for posttraumatic stress disorder (PTSD)^1–3^. Despite the promise of TF-CBT, as many as one-half of patients do not enjoy remission after therapy^4–6^. Accordingly, there is a need to understand why some patients are more responsive than others to TF-CBT. In response to this need, a series of studies have investigated neural markers that can predict response to TF-CBT^7–12^. Overall, these studies have found that better response to TF-CBT is associated with higher activation in the rostral anterior cingulate and posterior cingulate cortex during emotional processing and in the inferior parietal lobe and inferior frontal cortex during inhibitory tasks. Also, lower activation in the amygdala, insula, and dorsolateral prefrontal cortex during emotional and cognitive tasks is associated with a positive response^13^. Structural imaging studies have reported that larger hippocampal and rostral anterior cingulate volume prior to treatment predicts better treatment response^14,15^.

Much less attention has been given to the extent to which the white matter microstructure corresponding to neural tracts can predict response to TF-CBT. Brain white matter tracts are composed of nerve fibers or axons, which are the conduits for the transmission of electrical nerve signals and essential communication or connectivity between the functional gray matter regions of the brain. Diffusion-weighted imaging aims to evaluate this architecture at the macroscopic level by indirectly measuring the diffusion of water molecules along with different directions within a given voxel in the brain. Fractional anisotropy (FA) is a metric that captures this diffusion anisotropy and is considered to be an index of white matter organization^16^. Many studies have examined the properties of white matter in people with PTSD, with a recent meta-analysis of 30 studies comprising almost 450 adults and 300 children with PTSD showing different alterations in those with childhood and adult trauma^17^. This review showed that PTSD following adulthood trauma is associated with both higher and lower FA in the anterior and posterior part of the cingulum, superior longitudinal fasciculus, and frontal regions; PTSD following childhood trauma was associated with lower FA in the corpus callosum. One study has previously reported that lower FA values in the dorsal cingulum bundle, the main white matter tract connecting the anterior cingulate cortex, at baseline predict symptom reduction in PTSD following TF-CBT^18^. This finding accords with proposals that the anterior cingulate cortex is important for TF-CBT because it is implicated in emotion regulation and extinction learning^19^, and with evidence that its volume and activation recruitment prior to TF-CBT predicts better treatment response^8,9^.

The paucity of studies mapping the extent to which white matter microstructure is associated with response to TF-CBT in PTSD patients points to the need for further study. Additionally, recent evidence from functional imaging studies suggests that there could be differential neural predictors of improvement of the specific types of PTSD symptoms following TF-CBT^20^. While there is evidence that diffusion measures of white matter are associated with PTSD symptoms^21^, no previous study has evaluated if brain white matter structure is differentially associated with different PTSD symptoms following treatments. Accordingly, the aim of this study was to investigate the extent to which white matter FA prior to treatment is associated with symptom reduction in PTSD patients who receive a course of TF-CBT. In recognition that PTSD is a highly heterogeneous syndrome that does not reflect a uniform biological system^22^, we adopted the approach in this study of investigating the reduction of two subtypes of PTSD symptoms. Factor analytic studies of PTSD have shown that PTSD symptoms can be explained by two latent factors that comprise *fear* symptoms (including reexperiencing, active avoidance, hypervigilance, and elevated startle) and *dysphoric* symptoms (passive avoidance, sleep disturbance, concentration difficulties, and irritability) symptoms^23,24^. The rationale for recognizing these distinct subtypes is underscored by evidence that they can involve different neural networks; whereas fear symptoms are more likely to engage amygdala-dorsal ACC and dorsal medial prefrontal networks^25^, dysphoria symptoms can also implicate positive affect and reward circuitry^26^. We hypothesized that FA for white matter tracts related to the limbic amygdala-anterior cingulate brain regions i.e., the cingulum, fornix, stria-terminalis, and uncinate fasciculus to be associated with improvement of fear PTSD symptoms, whereas white matter tracts related to the cortico-basal ganglia-thalamocortical and dopaminergic reward pathways, for example, the anterior corona radiata, thalamic projections, external and internal capsule, to be associated with improvement of dysphoric PTSD symptoms.

## Materials and method

### Participants

Participants were recruited from one of two treatment trials between 2011 and 2016^27^. Only patients randomized to the TF-CBT arms of the two trials (determined by a random number generator on a 1:1 basis by researchers independent of the trial) were included in the current study. Of the 156 treatment-seeking PTSD patients eligible for the two trials, 84 were randomly allocated to the TF-CBT arm of which 51 consented for MRI scanning. Eleven of these individuals were lost to post-treatment assessments and a further four individuals were excluded due to poor MRI data quality (see Supplementary Fig. 1). The 36 patients (18 females, 18 males) included in the analysis had a mean age of 41.2 ± 11.2 years and had developed PTSD following the assault, childhood abuse, motor vehicle accidents, or police duties which occurred on average 36.3 ± 32.1 months prior to treatment. PTSD was diagnosed by clinical psychologists using the Clinician-Administered PTSD Scale (CAPS)^28^; a symptom is endorsed following the “2/1” method, indicating at least “moderate” distress and frequency of at least “once or twice a month”^28^. Participants with a history of neurological disorder, moderate or severe traumatic brain injury, psychosis, bipolar disorder, or substance dependence were excluded. The protocol permitted prescribed medication if the dosage had remained stable for two months prior to the scan and was not altered during the course of the study; 10 (27.8%) participants were taking selective serotonin reuptake inhibitors (SSRI). The study also included a comparison group of 33 healthy participants (17 females, 16 males) of mean age 38.2 ± 11.3 years who had never experienced a criterion A trauma and with no current Axis I disorders, as assessed by the Mini International Neuropsychiatric Interview (MINI version 5.5)^29^. Healthy participants were used only to compare pre-treatment white matter measures with the PTSD cohort and did not undergo a full clinical workup including the CAPS or TF-CBT as the PTSD cohort. Depression and anxiety levels were also assessed by self-report on the Depression, Anxiety, and Stress Scale (DASS)^30^. Participant characteristics are described in Table 1.

**Table 1:** Participant characteristics.

	PTSD	Controls
	(*n* = 36)	(*n* = 33)
Age, mean (*SD)*	41.2 (11.1)	38.2 (11.3)
Male, *n* (%)	18 (50.0)	16 (48.5)
Time since trauma, months, mean (*SD*)	36.3 (32.1)	–
Type of trauma, *n* (%)		
Childhood abuse	4 (11.1)	–
Motor vehicle accident	8 (22.2)	–
Police-related trauma	11 (30.6)	–
Assault	13 (36.1)	–
Prescribed SSRI, *n* (%)	10 (27.8)	–
Major depressive disorder, *n* (%)	20 (55.6)	–
Social phobia, *n* (%)	1 (2.8)	–
Panic disorder, *n* (%)	3 (8.3)	–
Agoraphobia, *n* (%)	0 (0)	–
Generalized anxiety disorder, *n* (%)	6 (16.7)	–
Obsessive-compulsive disorder, *n* (%)	0 (0)	–
DASS depression, mean (*SD*)	10.97 (4.85)	1 (1.52)
DASS anxiety, mean (*SD*)	8.25 (4.40)	0.85 (1.46)
Baseline CAPS severity, mean (*SD*)	72.3 (18.2)	–
Post-treatment CAPS severity, mean (*SD*)	28.8 (20.3)	–
% Change in CAPS severity	59.66 (30.8)	–
Pre-treatment dysphoria, mean (*SD*)	32 (11.5)	–
Post-treatment dysphoria, mean (*SD*)	14.5 (11)	–
% Change in dysphoria	56.38 (38.1)	–
Pre-treatment fear, mean, (*SD*)	39.7 (9.8)	–
Post-treatment fear, mean, (*SD*)	14.2 (11.4)	–
% Change in fear	64.27 (28.8)	–

Standard deviations appear in parentheses.

*CAPS* Clinician-Administered PTSD Scale, *PTSD* posttraumatic stress disorder, *SSRI* selective serotonin reuptake inhibitors.

### Procedure

The study was approved by the Western Sydney Area Health Service Human Research Ethics Committee and written informed consent was obtained from participants. The study procedure involved PTSD participants completing clinical and MRI assessments at baseline, followed by a course of TF-CBT and a posttreatment clinical assessment. Participants were initially assessed for PTSD (as defined by DSM-IV) by clinical psychologists using the CAPS and for comorbid Axis I disorders using the MINI including current major depressive episode, generalized anxiety disorder, social phobia, panic disorder, agoraphobia, obsessive-compulsive disorder, and a substance use disorder. Within two to three weeks of scanning, participants commenced a course of nine once-weekly individual 60–90 min sessions of TF-CBT that were delivered by experienced doctoral-level or masters-level clinical psychologists. Therapy involved an initial session of psychoeducation about psychological responses to trauma, then six sessions of 40-min imaginal exposure to the trauma memory, instructions regarding in vivo exposure to avoided situations, and cognitive restructuring of thoughts related to the traumatic event. An additional session reinforced cognitive restructuring exercises, and a final session focused on relapse prevention^27^. This therapy procedure is consistent with gold standard TF-CBT protocols^31^ although some treatment protocols allow up to 12 sessions of therapy, many require a minimum of nine sessions. Independent clinicians rated the fidelity of 130 sessions (18%), indicating full adherence to the treatment protocols and a high-level of quality on a seven-point scale (mean = 6.11, *SD* = 1.32). There were no treatment effects according to a different therapist or therapist qualifications. A posttreatment assessment using the CAPS was conducted by an independent clinical psychologist one week following completion of the course of treatment.

The change in PTSD symptom severity across treatment was calculated by subtracting post-treatment CAPS scores from pre-treatment scores. This score then was divided by the pretreatment CAPS score, to produce a CAPS change score independent of initial symptom severity. This change in PTSD severity score was used for correlation with white matter FA measures. To examine the change in PTSD severity, whilst considering the heterogeneity of the disorder, change in CAPS factor scores (fear factor scores and dysphoria factor scores) were also calculated in the same way as the overall CAPS-change scores, and were correlated with neural measures. The fear factor was defined as a total score of re-experiencing, active avoidance, hypervigilance, and elevated startle symptoms, and dysphoric factor as a total score of passive avoidance, sleep disturbance, concentration difficulties, and irritability symptoms.

### Imaging acquisition

All magnetic resonance imaging was performed on a 3.0 T GE Signa HDx scanner (GE Healthcare, Milwaukee, WI) using an eight-channel head coil. A spin-echo DTI–echo-planar imaging sequence was used to acquire diffusion-weighted images. Seventy contiguous, axial, 2.5-mm-thick slices (providing whole-brain coverage) were acquired in 42 gradient directions with a *b*-value of 1250 s/mm^2^. The imaging parameters were as follows: repetition time = 17,000 ms; echo time = 95 ms; fat saturation = ON; number of excitations = 1; frequency direction = right/left; in-plane resolution = 1.72 mm × 1.72 mm; 128 × 128 matrix. Four baseline (*b* = 0) images were also acquired at the start of the sequence.

All images were preprocessed, including skull stripping, eddy current correction, and head movement correction using FDT (FMRIB’s Diffusion Toolbox), available as part of the FSL software (https://fsl.fmrib.ox.ac.uk/) (version 5.0.9)^32^. A binary brain mask was created using the baseline non-diffusion-weighted image and diffusion tensor models were fitted for each brain voxel. FA, mean diffusivity (MD), and first, second, and third eigenvalue images were generated for each participant. Using tract-based spatial statistics (TBSS), all participant FA images were registered to the standard FMRIB58_FA template and transformed to Montreal Neurological Institute 152 1 mm^3^ standard space using nonlinear registrations^33^. After, all images were transformed, an average FA image was generated and then thinned to create a white matter skeleton comprising centers of white matter tracts that were common to all participants. The FA skeleton was set at a threshold level of FA ≥ 0.2, such that only major white matter pathways would be included. Next, every participant’s FA was projected onto the mean FA skeleton where each skeleton voxel was assigned a maximum value observed perpendicular to the tract.

Voxelwise associations of FA with the change in overall CAPS scores as well as specific fear and dysphoria symptoms scores were conducted on the skeletonized data for the PTSD patient group using “randomize” permutations testing available through the FSL software^34^. Permutations testing was set at 5000 permutations using the threshold-free cluster enhancement option^35^. To correct for multiple comparisons, the cluster-level correction was set at *α* < 0.05. To label clusters with significant FA associations, the John Hopkins University International Consortium for Brain Mapping 81 white-matter labels atlas was used^36^. To extract values for each tract, a binary mask was generated for each label cluster and the average FA was estimated for each participant for each label cluster. To further characterize significant FA changes, radial diffusivity (RD), axial diffusivity (AD), and MD diffusion parameters were also examined. The non-FA maps were projected onto the skeleton using the same parameters as for the FA images. The cluster binary mask in each label was used to extract values for AD (represented by λ1), RD (represented by the average value of λ2 and λ3), and MD and were used to test associations with symptom change measures using Pearson correlations in SPSS statistical software (IBM SPSS Statistics for Windows, Version 25.0. Armonk, NY: IBM Corp).

We then evaluated the neural measures significantly associated with the change scores between the PTSD cohort relative to the healthy controls by comparing mean FA for the significant clusters in SPSS. This was to evaluate whether the measures associated with response also characterized PTSD from controls.

As some participants (*n* = 10) in our PTSD group were currently on SSRI medications, we also evaluated whether there were any confounds from current medication use on the significant neural measures by re-running correlations between FA and symptom change scores in the PTSD cohort including medication use as a covariate. For all the secondary analyzes, we controlled multiple testing using the Benjamini-Hochberg FDR corrected *α* < 0.05 for statistical evaluation.

## Results

### Clinical outcomes

All 36 included participants met DSM-IV criteria for PTSD prior to treatment, and all participants completed seven to nine sessions of TF-CBT. The mean CAPS score was 72.3 ± 18.2 at pretreatment and 28.8 ± 20.3 following TF-CBT treatment. Participant characteristics are outlined in Table 1. PTSD participants had significantly higher DASS depression and anxiety levels compared to controls (*p* < 0.05).

### Voxel-wise whole-brain FA correlations with PTSD symptom improvement

**Fig. 1:**
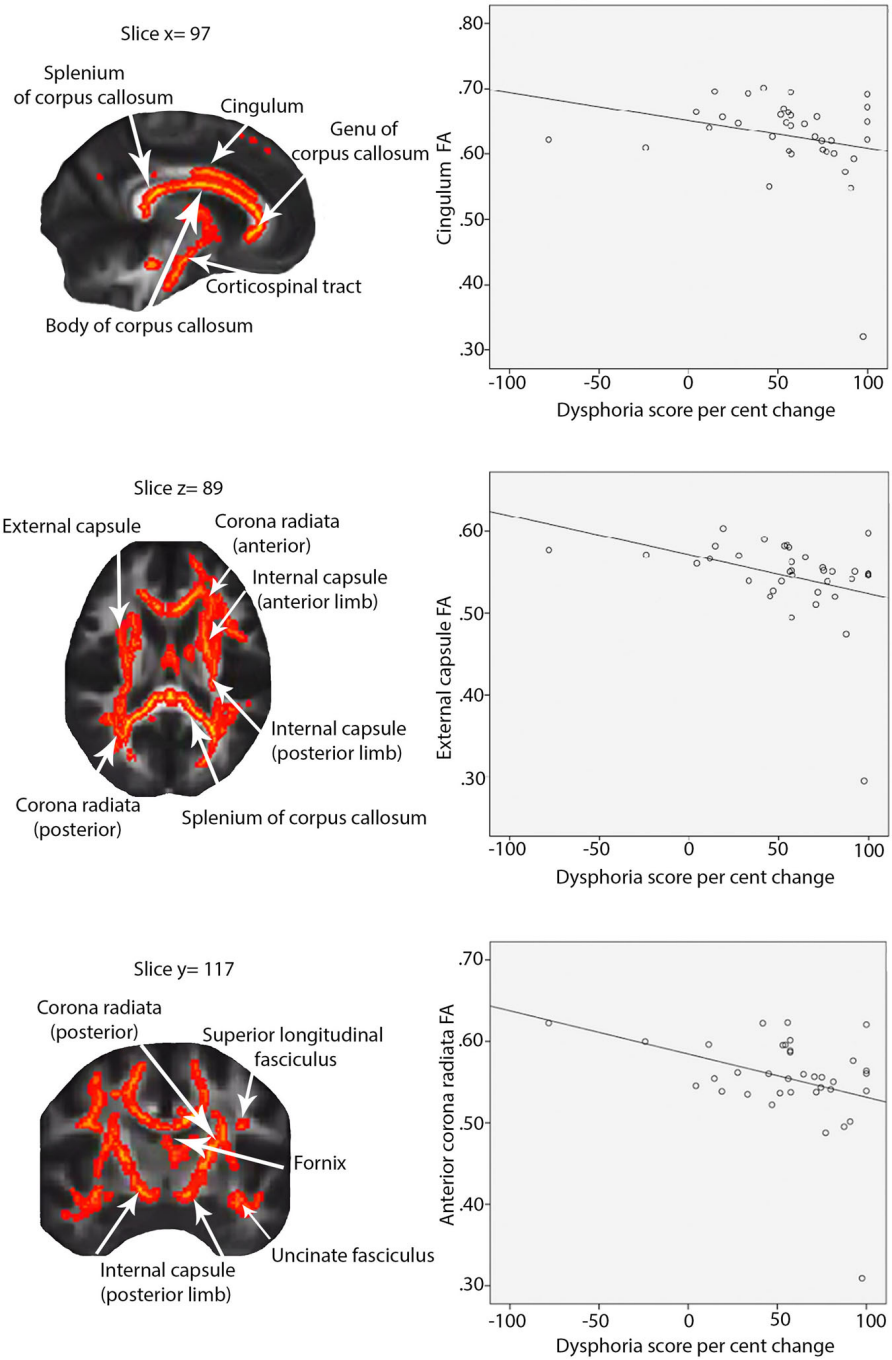
Significant white matter clusters for correlation between change in dysphoria score and FA. Clusters are overlaid on a mean fractional anisotropy image. Scatter plots indicate the relationship between FA in a few selected white matter regions with the change in dysphoria PTSD symptoms.

FA was significantly associated with improvement in dysphoria symptoms. Greater improvement in dysphoria following treatment was associated with lower values of FA in a number of white matter tracts prior to treatment (see Table 2). These included white matter regions associated with (1) the limbic regions i.e., bilateral cingulum bundle (cingulate portion), fornix, stria-terminalis, and uncinate fasciculus; (2) the interhemispheric bundles i.e., genu, body, and splenium of the corpus callosum; (3) projection fibers i.e., bilateral anterior, posterior and superior corona radiate, retrolenticular part of the internal capsule and posterior thalamic radiation; (4) association fibers i.e., bilateral sagittal stratum, external capsule, anterior and posterior limb of the internal capsule, superior longitudinal fasciculus, and superior fronto-occipital fasciculus; and (5) brainstem tracts i.e., bilateral corticospinal tract, cerebral peduncle, superior cerebellar peduncle, middle cerebellar peduncle, right inferior cerebellar peduncle, right medial lemniscus, and pontine crossing tract. Figure 1 shows selected slices depicting significant clusters overlaid over the white matter skeleton and standardized FA image. After controlling for SSRI medication use, all tracts remained significantly associated with the change in dysphoria scores. Of the above tracts, only the left posterior corona radiata FA was found to be different between the PTSD and controls groups (*p* = 0.04), however, this finding did not survive after FDR correction for multiple comparisons. There were no significant associations between FA and change in the fear scores or for overall CAPS scores.

**Table 2:** White matter regions with significant fractional anisotropy associations with dysphoria symptom change.

White-matter cluster	PTSD participants		Control participants		Correlation with change in dysphoria symptoms (*R*)	PTSD vs. control difference, %
	Mean FA	SD	Mean FA	SD		
**Limbic fibers**						
Left cingulum (cingulate gyrus)	0.63	0.07	0.64	0.04	−0.23	−0.76
Right cingulum (cingulate gyrus)	0.64	0.06	0.64	0.04	−0.099	0.68
Left fornix/stria-terminalis	0.56	0.06	0.55	0.05	−0.19	0.41
Right fornix/stria-terminalis	0.58	0.05	0.58	0.04	−0.132	0.24
Fornix body column	0.52	0.11	0.55	0.08	0.202	−3.55
Left uncinate fasciculus	0.58	0.07	0.57	0.05	−0.222	0.69
Right uncinate fasciculus	0.61	0.07	0.6	0.04	−0.141	0.42
**Corpus callosum**						
Genu	0.76	0.07	0.77	0.03	−0.25	−0.54
Body	0.68	0.06	0.69	0.04	−0.152	−0.83
Splenium	0.82	0.06	0.82	0.03	−0.214	−0.19
**Projection fibers**						
Left anterior corona radiata	0.54	0.06	0.53	0.03	−0.234	0.26
Right anterior corona radiata	0.55	0.05	0.55	0.03	−0.375	0.8
Left superior corona radiata	0.53	0.04	0.52	0.03	−0.279	0.73
Right superior corona radiata	0.53	0.05	0.53	0.03	−0.228	0.46
Left posterior corona radiata	0.57	0.04	0.55	0.03	−0.19	1.71^a^
Right posterior corona radiata	0.55	0.04	0.54	0.03	−0.171	1.12
Left retrolenticular part of internal capsule	0.62	0.05	0.61	0.03	−0.196	0.9
Right retrolenticular part of internal capsule	0.63	0.06	0.62	0.03	−0.155	0.99
Left posterior thalamic radiation	0.65	0.06	0.65	0.03	−0.194	0.18
Right posterior thalamic radiation	0.66	0.06	0.66	0.04	−0.169	−0.61
**Association fibers**						
Left sagittal stratum	0.6	0.05	0.59	0.03	−0.224	0.55
Right sagittal stratum	0.61	0.05	0.61	0.03	−0.133	−0.11
Left external capsule	0.54	0.05	0.54	0.03	−0.354	0.06
Right external capsule	0.52	0.05	0.52	0.03	−0.304	0.43
Left anterior limb of internal capsule	0.64	0.07	0.64	0.03	−0.335	−0.54
Right anterior limb of internal capsule	0.64	0.05	0.64	0.03	−0.427	0.53
Left posterior limb of internal capsule	0.71	0.07	0.7	0.03	−0.291	0.8
Right posterior limb of internal capsule	0.73	0.05	0.72	0.03	−0.272	0.92
Left superior longitudinal fasciculus	0.58	0.06	0.58	0.02	−0.216	−0.35
Right superior longitudinal fasciculus	0.56	0.05	0.56	0.03	−0.228	0.54
Left superior fronto-occipital fasciculus	0.54	0.06	0.55	0.05	−0.363	−0.02
Right superior fronto-occipital fasciculus	0.55	0.06	0.55	0.05	−0.13	0
**Tracts in the brainstem**						
Left corticospinal tract	0.68	0.06	0.66	0.03	−0.185	1.99
Right corticospinal tract	0.66	0.05	0.67	0.04	−0.3	−1.18
Left cerebral peduncle	0.75	0.06	0.75	0.03	−0.226	−0.35
Right cerebral peduncle	0.75	0.06	0.75	0.02	−0.238	0
Left superior cerebellar peduncle	0.78	0.06	0.77	0.04	−0.162	1.4
Right superior cerebellar peduncle	0.73	0.06	0.73	0.03	−0.248	0.58
Middle cerebellar peduncle	0.72	0.08	0.72	0.03	−0.185	−0.34
Right medial lemniscus	0.69	0.06	0.7	0.03	−0.139	−0.3
Right inferior cerebral peduncle	0.62	0.07	0.61	0.04	−0.138	0.64
Pontine crossing tract	0.57	0.06	0.57	0.04	−0.444	0.33

^a^Indicates significant difference between PTSD vs. Controls at *p* = 0.05.

*R* = correlation coefficient between fractional anisotropy and dysphoria symptoms change in the PTSD group (%).

### Correlations with diffusion measures

For the white matter regions reported above, positive correlations with dysphoria scores were observed for RD (the right external capsule: *r* = 0.339, *p* = 0.043), AD (left superior corona radiate: *r* = 0.419, *p* = 0.011), and MD (left superior corona radiata (*r* = 0.403, *p* = 0.015) and right anterior corona radiata (*r* = 0.352, *p* = 0.035)). However, none of these survived after adjusting for multiple comparisons.

## Discussion

This study investigated whether white matter FA prior to treatment is associated with symptom reduction in PTSD patients who receive a course of TF-CBT. While white matter FA was not found to be significantly associated with improvement of PTSD symptoms overall or fear symptoms, it was found to be associated with PTSD dysphoria symptom improvement. Lower pre-treatment FA in a number of white matter regions was associated with greater reductions in PTSD dysphoria symptoms. This highlights the heterogeneous nature of PTSD and the need to consider PTSD beyond a unitary construct especially in understanding mechanisms of treatment response.

A review of previous fMRI treatment studies in PTSD found functional activation in the anterior cingulate and amygdala to be most commonly associated with psychotherapy response^13^. Our study extends these functional findings by reporting the white matter structures associated with both these regions to be also associated with response to TF-CBT. The cingulum bundle and the fornix and stria-terminalis are outflow tracts linked to the anterior cingulate and amygdala/hippocampal limbic subcortical regions, respectively, and are involved in the formation of emotional memory, fear, and anxiety which are core symptom features of PTSD^37–39^. Similarly, the fornix connects the hippocampus to the hypothalamus, a key limbic structure that controls autonomic responses, whereas the stria-terminalis comprises connections between the amygdala and the bed nucleus of the stria-terminalis, a structure that orchestrates emotional and behavioral responses to stress through reciprocal connections to the cortical, midbrain, and brainstem regions, which are known to be involved in reward processes via the dopaminergic pathways^40,41^. The uncinate fasciculus also anatomically connects the anterior cingulate and amygdala regions^42^. Our findings seem to suggest that reduced FA in the above pathways may assist individuals in dampening their emotional responses to traumatic memories and their interference with reward processing, hence assisting them to benefit from TF-CBT and improving dysphoric symptoms. However, it is important to note that FA is a local measure of white matter microstructure orientation that is confined within the image voxel. Although FA reflects differences in some aspects of connectivity, it is difficult to interpret the direction of change i.e., lower or higher connectivity based only on FA^43^. Nevertheless, these findings are in line with the only other existing study that evaluated the effects of TF-CBT on brain white matter in veterans with PTSD^44^. This study also found that individuals with PTSD who remitted following TF-CBT treatment were characterized by lower FA in the right cingulum bundle both prior to and at 6 months following treatment relative to patients with persistent PTSD. However, in contrast, this same study failed to observe any differences between these patient groups in either the fornix or stria-terminalis at either baseline or following treatment. It is important to note that this study evaluated overall remission of PTSD rather than specific symptoms.

Although we had hypothesized these tracts to be primarily associated with improvement of fear symptoms, we found associations with the improvement of dysphoric symptoms. As hypothesized we also found associations with FA for tracts associated with the cortico-basal ganglia-thalamocortical reward brain pathways, including the internal and external capsule which links to the dorsal striatal brain regions and the thalamus, and the posterior thalamic radiation which links the thalamus and cortical brain regions^45^. The dysphoric phenotype of PTSD overlaps with depressive symptoms. In this context treatment studies in depression have also found FA values for the cingulum bundle and fornix/stria-terminalis to be associated with remission of depressive symptoms following antidepressant medication treatments^46^. This study also found lower FA in the stria-terminalis (but higher FA in the cingulum bundle) prior to treatment to be associated with depressive symptom remission. Another study in depressive individuals found that failure to remit to sertraline medication was associated with higher frontal FA values specifically in the superior frontal gyri and anterior cingulate cortices^47^. This might suggest that there are core neural features that are associated with depressive symptom improvement following treatment irrespective of the specific diagnosis.

We also observed a number of projection and association fiber bundles linked to the lateral prefrontal and parietal cortices to be associated with treatment response. For example, the superior longitudinal fasciculus and the superior fronto-occipital fasciculus, which connect some of the prefrontal cortical regions^45^, whereas the sagittal stratum which contains the inferior fronto-occiptal fascicle which is crucial for intra-hemispheric transfer of information between the frontal cortex and the occipital, temporal and parietal cortices^48^. The anterior corona radiata is part of the limbic-thalamo-cortical circuitry which includes thalamic projections from the internal capsule to the prefrontal cortical regions associated with impaired top-down emotion regulation systems in PTSD^45,49^. One of the main tenets of TF-CBT is the capacity to reappraise events and responses associated with the traumatic experience, such that they are experienced with less anxiety^50^. The cognitive control brain regions, specifically the dorsolateral prefrontal cortex, dorsal anterior cingulate, and parietal cortices, all play an important role in this process^51^. Our findings suggest that the “hard-wired” white matter *structural* connections associated with this *functional* network may also be crucial incapacity to learn skills to reappraise traumatic experiences and hence respond to treatment. However, more research is required to understand the direction of effects i.e., why lower FA in these white matter tracts was associated with greater improvement in symptoms. One possible avenue is to evaluate the longer-term impact on white-matter because of TF-CBT to understand how this relationship is mediated depending on treatment outcome. One previous study has demonstrated that white matter FA was found to increase as well as decrease as a function of response to treatment^44^. An increase in FA has been reported after learning^52^ and one could speculate that PTSD individuals who ultimately remit are the ones who have lower FA but demonstrate increased myelination effects and FA due to reappraisal related learning, whereas persistent PTSD individuals who already have a higher FA prior to treatment are unable to employ this plasticity and hence lack to benefit from TF-CBT. We also observed associations with FA in the corpus callosum and some of the tracts of the brainstem. The corpus callosum has previously been reported to be altered in PTSD^17,53^ and our study provides new evidence of its role in response to TF-CBT. Both the corpus callosum and some of the major tracts of the brainstem (e.g., corticospinal tract and the cerebral penduncles) are known to mediate connections between the cortical brain regions and between the cortex, thalamus, and brainstem, respectively, and hence could be likely associated with treatment response via the roles of these innervated regions.

In our analysis, the FA markers that predicted change in dysphoria symptoms were not found to be distinct between PTSD and controls. FA values for some of the tracts (e.g., the cingulum, anterior corona radiate, and superior longitudinal fasciculus) identified in our study have been previously reported as both increased as well as decreased in relation to PTSD^17^. However, a meta-analysis conducted in this study failed to identify any white matter FA alterations in PTSD relative to both trauma and healthy controls. This also concurs with our previous analysis that also failed to find any FA alterations in PTSD^54–56^. It is likely that white matter alterations exhibit only in PTSD concurrent with traumatic brain injuries^54–56^. The nature and type of trauma could also play a significant role in how white matter is impacted. For example, for children with PTSD or adult-onset PTSD due to childhood trauma, FA in the corpus callosum has been found to be impacted whereas PTSD due to traumatic experience in adulthood results in changes in the cingulum, superior longitudinal fasciculus, and frontal brain regions^17^. It is also possible that the treatment response markers of PTSD symptoms could be different from those that characterize PTSD as an overall diagnosis.

A number of methodological limitations are noted. First, we did not include a wait-list comparison group, which means it is hard to differentiate associations with response to TF-CBT and spontaneous remission; however, the latter explanation would not be expected considering that the meantime since trauma exposure was three years and most spontaneous remission from PTSD occurs within the first 12 months. Second, we note the modest sample size and acknowledge that a larger sample would allow for closer examination of PTSD subtypes. This also precluded analysis of different types of trauma, and specifically childhood abuse, for which the relationship with PTSD is associated with alterations in diverse white matter tracts, including the corpus callosum and cingulum^57^. We note that our sensitivity analyzes indicated that removing the four participants who developed PTSD secondary to childhood abuse or controlling for trauma type did not influence the results. Also, an independent sample is necessary to validate findings. Third, a proportion of participants were taking antidepressant medication prior to their course of TF-CBT, and it is possible that antidepressants may interact with TF-CBT; however, the medication dosage was stabilized prior to commencement of therapy and the dosage was not altered throughout the study. We were also able to replicate our findings controlling for medication use. Fourth, although the TBSS approach allows the study of whole-brain white matter without the necessity of definitive prior hypotheses, it is limited in the ability to ascertain the precise architecture of the involved tracts. Future work could benefit from using tractography approaches with high angular resolution diffusion imaging. Fifth, this design did not include a trauma-exposed control group which precludes us from isolating the effects of PTSD from trauma exposure. Replication of the current study could usefully include both trauma-exposed and healthy non-exposed control groups to disentangle the relative effects of trauma exposure and PTSD. Further, the inclusion of a depressed group would allow the white matter integrity associated with the dysphoric phenotype of PTSD to be compared to that of depressed patients.

In summary, our study provides evidence that the white-matter microstructure of the brain is associated with improvement in dysphoric symptoms following trauma focused-CBT in PTSD. Some of the key white matter tracts identified are linked to fear extinction, emotion regulation, and reward functional brain networks. This supports that white matter microstructure corresponding to these networks may also be important to functional recovery following TF-CBT. Our findings also highlight the role of different neural mechanisms for the improvement of different symptom phenotypes of PTSD. Future studies should consider this heterogeneity when assessing neural mechanisms of different treatments.

## Supplementary information

Supplementary Section
