# Supplementary material for: White matter anisotropy and response to cognitive behavior therapy for posttraumatic stress disorder
Source: Transl Psychiatry. 2021 Jan 5;11:14. doi: 10.1038/s41398-020-01143-3 (PMC7791115; doi:10.1038/s41398-020-01143-3)
Supplement: Supplementary file 1 — Supplementary Section [file 41398_2020_1143_MOESM1_ESM.docx]

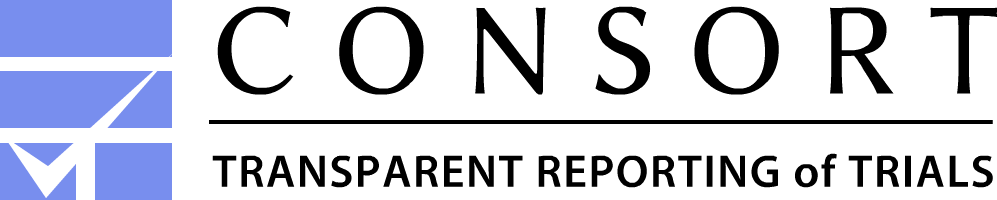


**Supplementary Figure S1: CONSORT 2010 Flow Diagram**

Allocated to TF-CBT (n= 84)

♦ Consented to MRI assessment (n = 51)

## Follow-Up

## Analysis

Not included in MRI study

Not included in MRI study

Lost to post-treatment assessment (n = 11)

## Enrollment

## Allocation

Allocated to Control conditions (n= 72)

♦ Consented to MRI assessment (n = 0)

Randomized (n= 156)

Excluded (n= 48)

♦  Not meeting inclusion criteria (n= 28 )

♦  Declined to participate (n=8)

♦  Other reasons (n=20 )

Assessed for eligibility (n= 204)

Completed post-treatment assessment (n = 40)
♦ Excluded from analysis because of MRI movement (n = 4)

♦  DTI data analysed (n=36)
